# Aortic arch branching variations and risk of cerebrovascular accidents in patients with a left ventricular assist device

**DOI:** 10.2459/JCM.0000000000001570

**Published:** 2023-12-08

**Authors:** Casper F. Zijderhand, Jelena Sjatskig, Denne A. Scharink, Jette J. Peek, Ozcan Birim, Jos A. Bekkers, Ad J.J.C. Bogers, Kadir Caliskan

**Affiliations:** a Thoraxcenter, Department of Cardiothoracic Surgery; b Department of Cardiology, Erasmus MC University Medical Center, Rotterdam, The Netherlands

**Keywords:** adverse events, anatomic variations, aortic arch branching, left ventricular assist device

## Abstract

**Aims:**

This retrospective study investigated the association between anatomical variations in the aortic arch branching and adverse events, including the risk of cerebrovascular accidents (CVAs), in patients with a left ventricular assist device (LVAD).

**Methods:**

Medical charts were reviewed for all patients with HeartMate 3 LVAD support at our center from 2016 to 2021. Computed tomography scans were evaluated to categorize the variations in the aortic arch branching based on seven different types, as described in the literature.

**Results:**

In total, 101 patients were included: 86 (85.1%) with a normal branching pattern and 15 (14.9%) with an anatomical variation. The following variations were observed: eight (7.9%) with a bovine arch and seven (6.9%) with a left vertebral arch. The median age was 57 years, 77.2% were men, and the median follow-up was 25 months. No difference was found in the rate of early (< 30 days) re-exploration due to bleeding after LVAD implantation. The rate of CVA and mortality did not differ significantly between patients with a normal arch or an anatomical variation during follow-up, with hazard ratios of 1.47 [95% confidence interval (CI): 0.48–4.48; *P* = 0.495] and 0.69 (95% CI: 0.24–1.98; *P* = 0.489), respectively.

**Conclusion:**

This preliminary study showed no differences in early and long-term adverse events, including CVA, when comparing patients with a variation in the aortic arch branching to patients with a normal aortic arch. However, knowledge of the variations in aortic arch branching could be meaningful during cardiac surgery for potential differences in surgical events in the perioperative period.

## Introduction

Cerebrovascular accidents (CVAs) are one of the leading complications and causes of mortality in patients with end-stage heart failure who are treated with a left ventricular assist device (LVAD).^1^ The rate of CVAs in patients with LVAD support is high; 10% of individuals are affected in the first year of support and the rate of CVAs continues to increase, up to 16% during follow-up.^1–3^ In prior studies, risk factors for CVAs during LVAD support have included atrial fibrillation, hypertension, more severe heart failure, female sex, diabetes mellitus, and a history of a CVA pre-LVAD implantation.^4–6^ However, other potential risk factors remain to be elucidated to decrease the risk of CVAs post-LVAD implantation.

One potential risk factor for CVAs during LVAD support could be the anatomical variations in the aortic arch branching given the close relationship with LVAD outflow graft insertion and flow in the ascending aortic. The aortic arch standard branching pattern consists of three main branches: the brachiocephalic trunk, which branches into the right common carotid artery and the right subclavian artery, followed by the left common carotid artery and the left subclavian artery.^7^ Several anatomical variations in the branching are known in the general population.^8^ Anatomical variants have been identified as potential risk factors for hemorrhage and ischemia during cardiac surgery, as indicated by a previous study.^8^ These variants could also increase the risk of left hemispheric laterality in cardioembolic CVAs, which are associated with worse outcomes compared with right laterality.^9,10^ LVAD-supported patients have an elevated risk of developing CVA, and this risk could potentially be influenced by anatomical variations in the aortic arch branching.

However, to the best of our knowledge, there are no reports regarding the potential influence of anatomical variations in the aortic arch branching on the risk of CVAs. Therefore, this study aimed to elucidate the potential risk of CVAs in LVAD patients with aortic arch branching variations.

## Materials and methods

### Study design and data collection

Hospital records were reviewed retrospectively for all patients who underwent a HeartMate 3 (Abbott, Chicago, Illinois, USA) implantation in the Erasmus Medical Center between January 2016 and December 2021. All LVAD implantations were performed through a median sternotomy and on extracorporeal circulation. All implantations were carried out by three different surgeons employing similar techniques for positioning the inflow cannula and the anastomosis of the outflow graft on the ascending aorta. Our anticoagulation protocol, according to the current LVAD guidelines, involves the use of Vitamin K antagonists (such as acenocoumarin) with a target INR value of between 2 and 3, combined with aspirin 80 mg daily. Occasionally, this may be interrupted or discontinued when a patient experiences major bleeding event(s). Patients were eligible if they underwent a contrast-enhanced computed tomography (CT) scan of the chest pre or post-LVAD implantation and were 18 years or older. Patient characteristics before LVAD implantation and procedural characteristics were collected from the local input of the European Registry for Patients with Mechanical Circulatory Support (EUROMACS) registry.

### Anatomical variations in the aortic arch branching

To evaluate the anatomical variation in the aortic arch branching, a contrast-enhanced CT scan of the chest pre or post-LVAD implantation was used, depending on availability. The chest CT scan was considered applicable if it contained the course of the aortic branch vessels. The anatomical variations were evaluated based on the coronal and axial planes. The anatomical variations were scored into seven different categories, as described in the literature: normal (Type 1), bovine (Type 2), left vertebral (Type 3), bovine and left vertebral (Type 4), common carotid (Type 5), aberrant right subclavian (Type 6), and right arch (Type 7).^8^

### Outcomes

The primary endpoint was the association between anatomical variations in the aortic arch branching and the occurrence of CVAs. Secondary endpoints were early renal failure, re-exploration for bleeding early after LVAD implantation, unexpected readmission, mortality, infection, and late bleeding. Early outcomes were defined as outcomes within the first 30 days after LVAD implantation and late outcomes were defined as 30 days or later after LVAD implantation.

### Statistical analysis

Baseline categorical data were presented as counts and percentages, and continuous variables were presented as mean and standard deviation (SD) if they were normally distributed, or as median with interquartile range (IQR) if they were nonnormally distributed. The normality was tested using the Shapiro–Wilk test. Differences between patients with or without anatomical variation were evaluated using the Student's *t*-test or Mann–Whitney *U*-test, depending on the distribution of the continuous variables. Differences between categorical variables were expressed as count and percentage and compared using the chi-squared test or Fisher's exact test (if any of the expected cell sizes was ≤5) to assess the association. Early clinical outcomes were calculated using logistic regression and presented as odds ratios (ORs). Differences in clinical outcomes over time were calculated and presented as hazard ratios using a Cox proportional hazard model. Kaplan--Meier curves were plotted to visualize primary and secondary outcomes, where applicable, and compared using the log-rank test. It should be noted that unexpected hospital readmission could occur multiple times in one patient, which could have affected the mean occurrence rates. To provide a comprehensive overview, considering multiple recurrent events, the cumulative mean number of events over time was calculated using a nonparametric mean cumulative function (MCF) and presented in a plot.^11^ The variance was estimated using the Lawless and Nadeau method.^12^ Statistical analyses were performed using R (Version 4.1.2).

## Results

### Patient population

Overall, 110 patients received a LVAD during the study period and were screened for anatomical variations in the aortic arch. In total, 101 patients met the requirements for analysis. The other nine patients were excluded since the aortic arch was not scanned extensively enough to identify the branching pattern. The median age was 57 years (IQR: 52–62), with 77.2% being men, and a median follow-up of 25 months (IQR: 10–37). The most frequent cause of heart failure was ischemic heart disease (52.5%), and the patients were mainly in INTERMACS (Interagency Registry for Mechanically Assisted Circulatory Support) profiles 3 and 4 before implantation. Atrial fibrillation was present in 19.4% of the patients, and 82.2% had an implantable cardioverter-defibrillator (ICD) in place. The most prevalent LVAD strategy was bridge-to-transplantation, with 63.4% of the patients, and destination therapy in 32.7%. The median cardiopulmonary bypass time (CPB) was 97 min (IQR: 81–119), and the median time in the operating room was 338 min (IQR: 287–416). The median length of ICU stay was 8 days (IQR: 5–17), and the median hospital stay was 29 days (IQR: 23–43) (Table 1).

**Table 1:** Baseline and procedural characteristics

	Overall *N* = 101	Normal arch *N* = 86	Variation *N* = 15	*P*
Demographics				
Age in years	57.0 [52.0, 62.0]	57.0 [52.0, 62.8]	59.0 [51.0, 62.0]	0.731
Male	78 (77.2)	67 (77.9)	11 (73.3)	1.000
BMI	23.0 ± 3.6	23.0 ± 3.5	23.0 ± 3.9	0.957
Primary diagnosis				
Ischemic heart disease	53 (52.5)	45 (52.3)	8 (53.3)	1.000
Nonischemic heart disease	48 (47.5)	41 (47.7)	7 (46.7)	
INTERMACS patient profile				
1	18 (17.8)	17 (19.8)	1 (6.7)	0.516
2	19 (18.8)	17 (19.8)	2 (13.3)	
3	27 (26.7)	22 (25.6)	5 (33.3)	
≥4	37 (36.6)	30 (34.9)	7 (46.7)	
Comorbidities				
Diabetes	24 (23.8)	20 (23.3)	4 (26.7)	1.000
ICD therapy	83 (82.2)	69 (80.2)	14 (93.3)	0.391
Neurological event	9 (9.0)	8 (9.4)	1 (6.7)	0.665
COPD	5 (5.0)	4 (4.6)	1 (6.7)	0.559
Preoperative status				
Intra-aortic balloon pump	26 (25.7)	24 (27.9)	2 (13.3)	0.384
Extracorporeal membrane oxygenation	8 (7.9)	8 (9.3)	0 (0.0)	0.437
Intravenous inotropes	55 (68.8)	56 (65.1)	10 (66.7)	1.000
Beta blockers	47 (46.5)	40 (46.5)	7 (46.7)	1.000
RAAS inhibitors	49 (48.5)	42 (48.8)	7 (46.7)	1.000
ECG rhythm				
Sinus	52 (53.1)	44 (53.0)	8 (53.3)	0.996
Atrial fibrillation	19 (19.4)	16 (19.3)	3 (20.0)	
Paced	27 (27.6)	23 (27.7)	4 (26.7)	
Procedural characteristics				
Bridge to transplant	64 (63.4)	54 (62.8)	10 (66.7)	0.694
Destination therapy	33 (32.7)	28 (32.6)	5 (33.3)	
Other	4 (4.0)	4 (4.7)	0 (0.0)	
Cardiopulmonary bypass time (min)	97.0 [81.0, 119.0]	98.0 [83.0, 128.0]	81.0 [76.0, 97.0]	0.052
Time in operating room for implant (min)	338.0 [287.0, 416.0]	339.0 [285.5, 416.3]	316.0 [298.5, 408.5]	0.992
ICU stay (days)	8.0 [5.0, 17.3]	8.0 [5.0, 18.0]	6.0 [4.5, 8.0]	0.111
Hospital stay (days)	29.0 [23.0, 43.0]	30.0 [23.0, 45.5]	25.0 [21.0, 29.5]	0.102
Length of follow-up (months)	25.0 [10.0, 37.0]	25.0 [10.0, 36.0]	28.0 [18.5, 43.5]	0.307

Continuous variables are described as mean and standard deviation (SD) or median [interquartile range (IQR)]. Categorical variables are described as count (percentage). ICD, implantable cardioverter-defibrillator; INTERMACS, interagency registry for mechanically assisted circulatory support; RAAS inhibitors, renin-angiotensin-aldosterone system inhibitors.

### Baseline characteristics

In total, 15 patients (14.9%) were identified with an anatomical variation: 8 (7.9%) with a bovine arch (Type 2) and 7 (6.9%) with a left vertebral arch (Type 3). The other anatomical variations were not observed in our patient population. No differences were found regarding the baseline characteristics in patients with or without an anatomical variation. CPB time showed a trend towards significance with a shorter time in patients with an anatomical variation [81 min (IQR: 76–97) vs. 98 min (IQR: 83–128); *P* = 0.052]. Time in the operating room for implant did not differ (respectively, 316 vs. 339 min; *P* = 0.992). The median follow-up time was comparable between patients with a normal arch and an anatomical variation (respectively, 25 vs. 28 months; *P* = 0.307) (Table 1).

### Outcomes

**Fig. 1:**
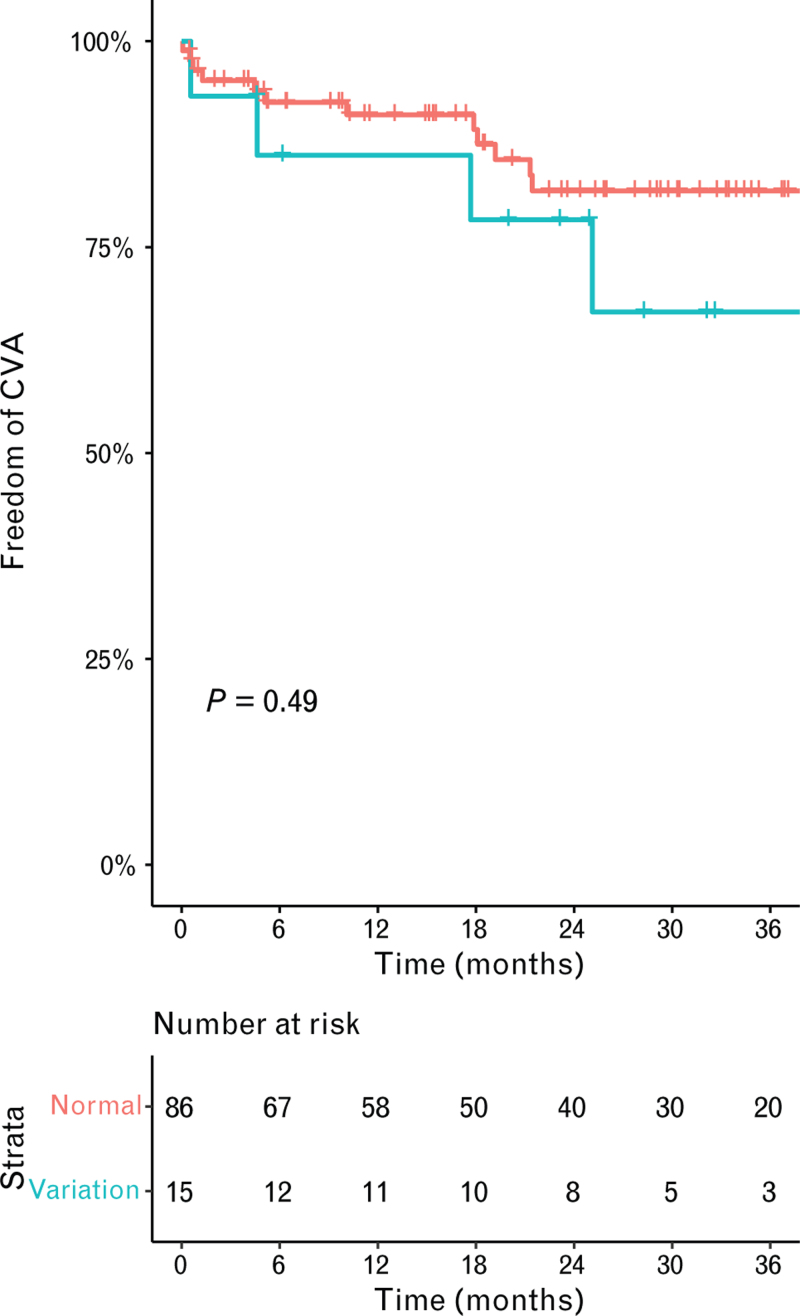
Kaplan--Meier estimates of cerebrovascular accidents stratified by normal anatomy (red line) and variation in the aortic arch branches (blue line). CVA, cerebrovascular accident.

**Fig. 2:**
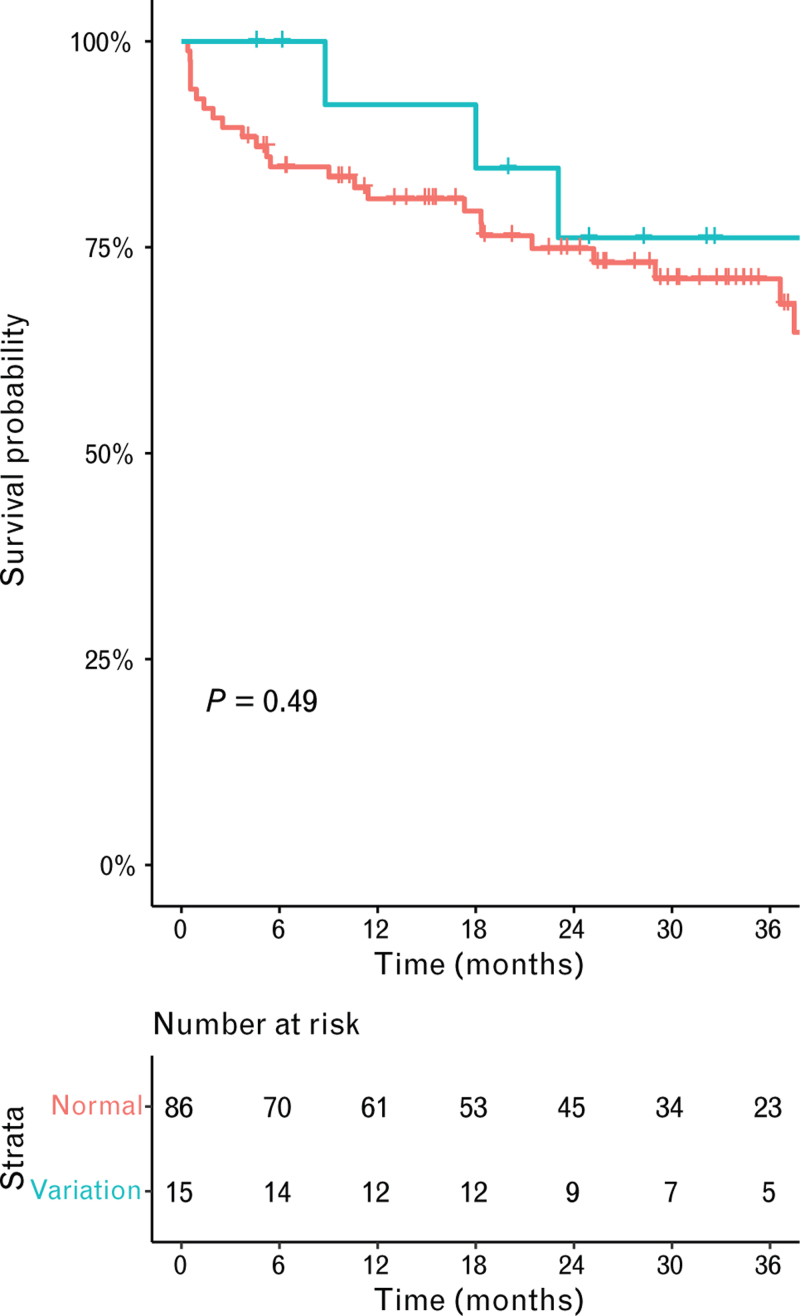
Kaplan--Meier estimates of survival stratified by normal anatomy (red line) and variation in the aortic arch branches (blue line).

**Fig. 3:**
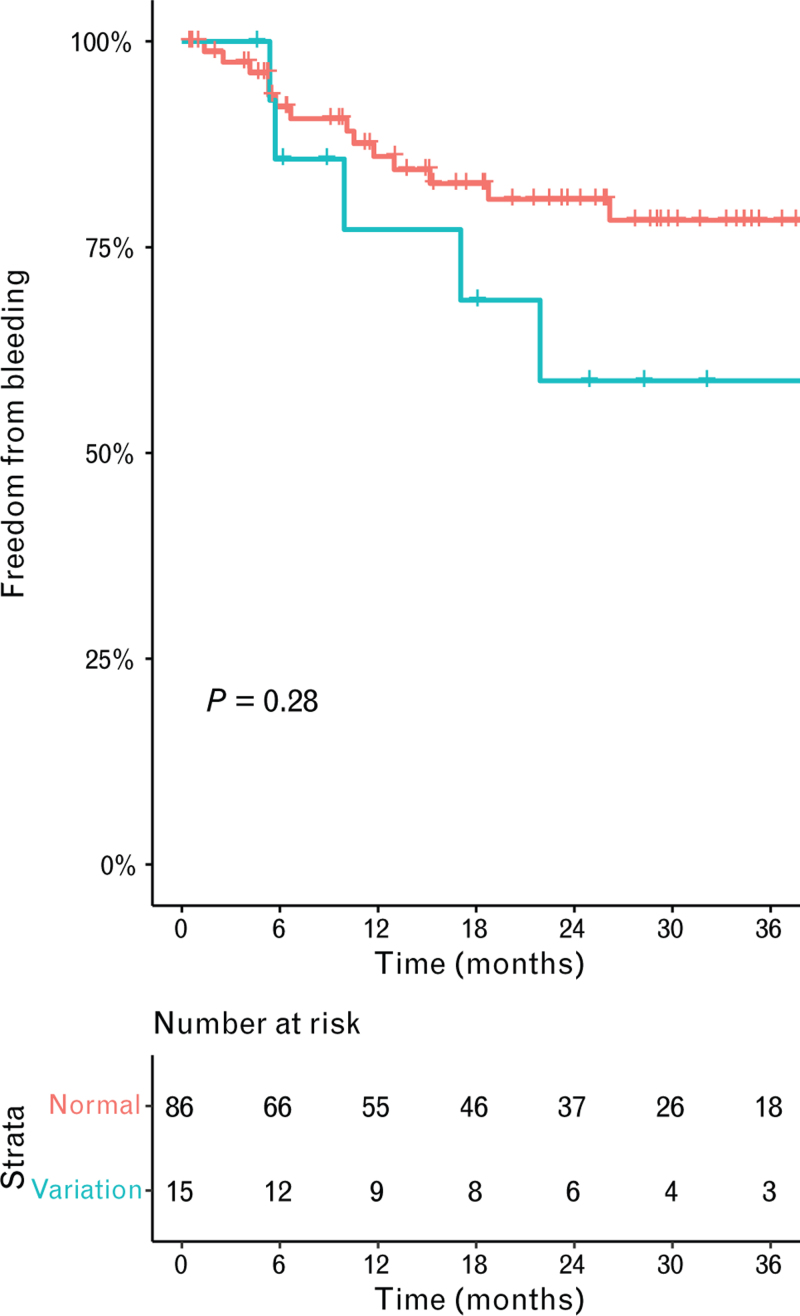
Kaplan--Meier estimates of bleeding stratified by normal anatomy (red line) and variation in the aortic arch branches (blue line).

**Fig. 4:**
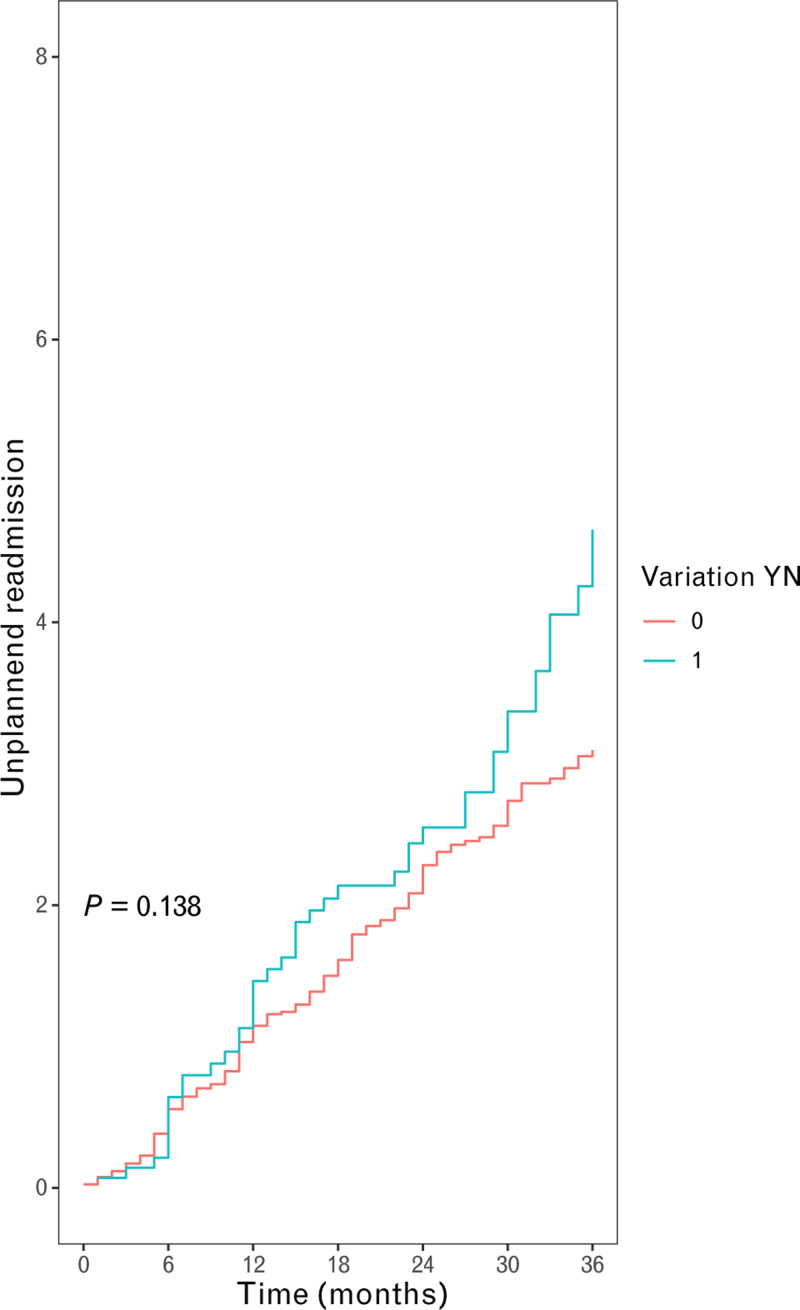
Mean cumulative function of unplanned hospital readmissions stratified by normal anatomy (red line) or variation in the aortic arch branches (blue line). The *y*-axis presents the number of recurrent unplanned hospital readmissions, and the *x*-axis represents the time in months.

Early acute kidney injury and the need for early surgical re-exploration for potential bleeding did not differ between the patients with or without anatomical variation, respectively, with an OR of 1.24 [95% confidence interval (95% CI): 0.39–3.79; *P* = 0.705] and an OR of 2.13 (95% CI: 0.70–6.64; *P* = 0.180, Table 2). The primary endpoint, the occurrence of CVAs, was comparable between patients with an anatomical variation, with a hazard ratio of 1.47 (95% CI: 0.48–4.48; *P* = 0.495, log-rank test *P* = 0.490; Fig. 1). In total, 18 patients suffered from a CVA during the follow-up, of whom 11 were ischemic and 7 were hemorrhagic. The laterality of the CVA was right hemispheric CVA in 10 patients and left hemispheric CVA in 8 patients. Secondary endpoints, survival, and late bleeding were comparable between patients with or without anatomical variation, respectively, with a hazard ratio of 0.69 (95% CI: 0.24–1.98; *P* = 0.489, log-rank test *P* = 0.490, Fig. 2) and a hazard ratio of 1.72 (95% CI: 0.63–4.71; *P* = 0.288, log-rank test *P* = 0.280; Fig. 3 and Table 2). Subsequently, infection during follow-up did not differ between with or without anatomical variation, with a hazard ratio of 0.61 (95% CI: 0.72–4.71; *P* = 0.199, Table 2). The number of unplanned readmissions over time did not show a significant difference during follow-up (3.4 vs. 2.2 in 36 months, *P* = 0.138; Fig. 4).

**Table 2:** Early and late outcomes in patients with a normal aortic arch branching pattern vs. patients with a variation in the aortic arch branching pattern

	Normal arch *N* = 86	Variation *N* = 15	OR/HR	95% CI	*P*
Early (<30 days)					
Acute kidney injury	30	6	1.24^a^	0.39–3.79	0.705
Surgical re-exploration for potential bleeding	30	8	2.13^a^	0.70–6.64	0.180
Late (>30 days)					
CVA	14	4	1.47	0.48–4.48	0.495
Survival	26	4	0.69	0.24–1.98	0.489
Bleeding	17	5	1.72	0.63–4.71	0.280
Infection	18	6	0.61	0.72–4.63	0.199

Data are presented as the number of events, odds ratio (OR) for early events (< 30 days), hazard ratio (HR) for late events (> 30 days), and 95% confidence interval (CI).

a Data are presented as odds ratio (OR).

## Discussion

In this study, we analyzed the association between anatomical variation in the branching of the aortic arch and cerebral thromboembolic events. No differences were observed between patients with normal anatomy and anatomical variation regarding the occurrence of CVAs, survival, and early adverse events, including acute kidney injury and surgical re-exploration for potential bleeding. Furthermore, the occurrences of late bleeding events and unplanned readmission were comparable. This suggests the negligible influence of anatomical variation in the branching of the aortic arch on postoperative adverse events during HeartMate 3 LVAD support in our study population.

### Anatomical variation in the aortic arch branching

According to the literature, the ‘normal’ aortic arch branching pattern (Type 1) is found with an incidence of 65--94% in the general population.^8,13^ Our study describes a relative incidence of 85.1%, which is consistent with earlier studies. Furthermore, we only observed patients with a bovine arch (Type 2) and a left vertebral arch (Type 3). Patients with a bovine arch are often asymptomatic, and this type of anatomical variation may rarely cause clinical symptoms. However, there may be an association with an increased risk in interventions for thoracic aortic disease.^14,15^ Patients with a left vertebral arch are also often asymptomatic. On the basis of a small series of patients, a left vertebral artery is suggested to be associated with an increased risk of postoperative bleeding after cardiac surgery.^16^ The other anatomical variations (Types 4 to 7) are rare, with an incidence of less than 1%.^8,13^ These variations were not observed in our study population, presumably due to the limited sample size.

### Cerebrovascular accidents

CVAs are common complications in LVAD patients during follow-up and remain the most common cause of death between 6 and 24 months after implantation.^1^ Our study did not reveal a difference in the occurrence of CVAs, both ischemic and hemorrhagic, during follow-up in patients with or without anatomical variation in the aortic arch branching. A small sample size study suggested an association between patients with a bovine arch and left hemispheric laterality due to a cardioembolic CVA in patients with atrial fibrillation.^10^ However, to the best of our knowledge, no other studies have been published reporting an elevated risk of CVAs in patients with anatomical variation in the aortic arch branching in the general population. In the general population, left hemispheric CVAs occur more frequently and are often associated with worse outcomes than right hemispheric CVAs.^9^ However, our data showed more right hemispheric and hemorrhagic CVAs during follow-up. This could be attributed to our small sample size compared with other studies that focused on CVAs laterality in larger population-based cohorts.

### Adverse events

Earlier studies have described a greater risk of hemorrhage and ischemia during cardiac surgery in patients with an anatomical variation in the aortic arch branching.^8^ In addition, prolonged CPB time appears to have adverse effects on clinical outcomes.^17^ However, our study did not find a significant difference in CPB time among patients with an anatomical variation. Furthermore, there were no differences in adverse events during follow-up, including surgical re-exploration for bleeding after LVAD implantation. These findings contradict previous research and highlight the need for further investigation with a larger sample size to reduce the possibility of type II errors. A recent study observed a significant association between variations in the branching of the aortic arch and thoracic artery diseases, such as aneurysmal dilatation or aortic dissection of the thoracic aorta.^14^ Earlier studies also described decreased survival in patients with thoracic artery disease.^18^ However, in our study, we found no difference in the survival rate during follow-up between patients with and without anatomical variation in the aortic arch branching. On the basis of the literature, we suggest that clinicians should be aware of and regularly evaluate patients with an anatomical variation in the aortic arch branching for thoracic artery disease to prevent potential adverse events, even after LVAD implantation.

## Limitations

It is important to acknowledge several limitations inherent to our retrospective observational study. Firstly, our results are constrained by a small sample size, which increases the risk of type II errors in the comparisons made. In addition, it should be noted that we did not observe Types 4 to 7 anatomical variations in the aortic arch branching in our study, thus limiting the generalizability of our conclusions to all possible variations. However, these types of variations are rare in the general population, and our study focused on describing the most common types to provide a comprehensive overview. Furthermore, no significant differences were found in postoperative adverse events between patients with and without anatomical variations. However, larger studies with a more substantial sample size are necessary to draw definitive conclusions regarding the relationship between adverse events and anatomical variation in the aortic arch branching among LVAD patients.

## Conclusion

This preliminary study indicates no differences in early and long-term adverse events, including CVAs, among patients who received a HeartMate 3 LVAD implantation and anatomical variations in the aortic arch branching. Nevertheless, awareness of variations in aortic arch branching remains important during cardiac surgery and in the long term due to the increased risk of aneurysmal dilatation and dissection associated with certain variations. Further research, ideally with a multicenter design and a larger sample size, is needed to establish more definitive conclusions.

## Acknowledgements

All authors have made substantial contributions to the conception or design of the manuscript, drafted or critically reviewed the manuscript, and approved the manuscript for publication.

General approval was obtained from the institutional medical ethical committee to conduct retrospective chart studies for potential LVAD complications and outcomes (MEC-2017–1013).

The authors received no specific funding for this work.

### Conflicts of interest

There are no conflicts of interest.

## References

[1] Kirklin JK Pagani FD Kormos RL. Eighth annual INTERMACS report: special focus on framing the impact of adverse events. *J Heart Lung Transplant* 2017; 36:1080–1086. 28942782 10.1016/j.healun.2017.07.005

[2] Acharya D Loyaga-Rendon R Morgan CJ. INTERMACS analysis of stroke during support with continuous-flow left ventricular assist devices: risk factors and outcomes. *JACC Heart Fail* 2017; 5:703–711. 28958345 10.1016/j.jchf.2017.06.014 PMC5743224

[3] Kirklin JK Naftel DC Myers SL Pagani FD Colombo PC. Quantifying the impact from a stroke during support with continuous flow ventricular assist devices: an STS INTERMACS analysis. *J Heart Lung Transplant* 2020; 39:782–794. 32376278 10.1016/j.healun.2020.04.006

[4] Antonides CFJ Yalcin YC Veen KM. Survival and adverse events in patients with atrial fibrillation at left ventricular assist device implantation: an analysis of the European Registry for Patients with Mechanical Circulatory Support. *Eur J Cardiothorac Surg* 2022; 61:1164–1175. 35076057 10.1093/ejcts/ezac023 PMC9070499

[5] Frontera JA Starling R Cho SM. Risk factors, mortality, and timing of ischemic and hemorrhagic stroke with left ventricular assist devices. *J Heart Lung Transplant* 2017; 36:673–683. 28110971 10.1016/j.healun.2016.12.010

[6] Kato TS Schulze PC Yang J. Preoperative and postoperative risk factors associated with neurologic complications in patients with advanced heart failure supported by a left ventricular assist device. *J Heart Lung Transplant* 2012; 31:1–8. 21986099 10.1016/j.healun.2011.08.014 PMC4945759

[7] Liechty JD Shields TW Anson BJ. Variations pertaining to the aortic arches and their branches; with comments on surgically important types. *Q Bull Northwest Univ Med Sch* 1957; 31:136–143. 13453649 PMC3803575

[8] Popieluszko P Henry BM Sanna B. A systematic review and meta-analysis of variations in branching patterns of the adult aortic arch. *J Vasc Surg* 2018; 68:298–306. e10. 28865978 10.1016/j.jvs.2017.06.097

[9] Hedna VS Bodhit AN Ansari S. Hemispheric differences in ischemic stroke: is left-hemisphere stroke more common? *J Clin Neurol* 2013; 9:97–102. 23626647 10.3988/jcn.2013.9.2.97 PMC3633197

[10] Matakas JD Gold MM Sterman J. Bovine arch and stroke laterality. *J Am Heart Assoc* 2020; 9:e015390. 32552234 10.1161/JAHA.119.015390 PMC7670536

[11] Nelson W. Recurrent events data analysis for product repairs, disease recurrences, and other applications Philadelphia: Society for Industrial and Applied Mathematics; 2003. http://epubs.siam.org/ebooks/siam/asa-siam_series_on_statistics_and_applied_probability/sa10.

[12] Lawless JF Nadeau C. Some simple robust methods for the analysis of recurrent events. *Technometrics* 1995; 37:158–168.

[13] Natsis KI Tsitouridis IA Didagelos MV Fillipidis AA Vlasis KG Tsikaras PD. Anatomical variations in the branches of the human aortic arch in 633 angiographies: clinical significance and literature review. *Surg Radiol Anat* 2009; 31:319–323. 19034377 10.1007/s00276-008-0442-2

[14] Dumfarth J Chou AS Ziganshin BA. Atypical aortic arch branching variants: a novel marker for thoracic aortic disease. *J Thorac Cardiovasc Surg* 2015; 149:1586–1592. 25802134 10.1016/j.jtcvs.2015.02.019

[15] Faggioli GL Ferri M Freyrie A. Aortic arch anomalies are associated with increased risk of neurological events in carotid stent procedures. *Eur J Vasc Endovasc Surg* 2007; 33:436–441. 17240174 10.1016/j.ejvs.2006.11.026

[16] Daentzer D Deinsberger W Boker DK. Vertebral artery complications in anterior approaches to the cervical spine: report of two cases and review of literature. *Surg Neurol* 2003; 59:300–309. discussion 9. 12748015 10.1016/s0090-3019(03)00113-7

[17] Nadeem R Agarwal S Jawed S Yasser A Altahmody K. Impact of cardiopulmonary bypass time on postoperative duration of mechanical ventilation in patients undergoing cardiovascular surgeries: a systemic review and regression of metadata. *Cureus* 2019; 11:e6088. 31857920 10.7759/cureus.6088 PMC6897343

[18] Sidloff D Choke E Stather P Bown M Thompson J Sayers R. Mortality from thoracic aortic diseases and associations with cardiovascular risk factors. *Circulation* 2014; 130:2287–2294. 25394733 10.1161/CIRCULATIONAHA.114.010890

